# Access to Technology and Preferences for an mHealth Intervention to Promote Medication Adherence in Pediatric Acute Lymphoblastic Leukemia: Approach Leveraging Behavior Change Techniques

**DOI:** 10.2196/24893

**Published:** 2021-02-18

**Authors:** Mallorie B Heneghan, Tasmeen Hussain, Leonardo Barrera, Stephanie W Cai, Maureen Haugen, Elaine Morgan, Jenna Rossoff, Joanna Weinstein, Nobuko Hijiya, David Cella, Sherif M Badawy

**Affiliations:** 1 Division of Pediatric Hematology/Oncology Department of Pediatrics University of Utah Salt Lake City, UT United States; 2 Division of Internal Medicine Northwestern University Feinberg School of Medicine Chicago, IL United States; 3 Mary Ann & J. Milburn Smith Child Health Research, Outreach, and Advocacy Center Ann & Robert H. Lurie Children’s Hospital of Chicago Chicago, IL United States; 4 Department of Obstetrics & Gynecology Northwestern University Feinberg School of Medicine Chicago, IL United States; 5 Division of Hematology, Oncology and Stem Cell Transplantation Ann & Robert H. Lurie Children's Hospital of Chicago Chicago, IL United States; 6 Department of Pediatrics Northwestern University Feinberg School of Medicine Chicago, IL United States; 7 Department of Pediatrics Columbia University Medical Center New York, NY United States; 8 Department of Medical Social Sciences Northwestern University Feinberg School of Medicine Chicago, IL United States

**Keywords:** acute lymphoblastic leukemia, medication adherence, behavior change technique, oral chemotherapy, mHealth, patient-centered

## Abstract

**Background:**

Suboptimal adherence to 6-mercaptopurine (6-MP) is prevalent in pediatric acute lymphoblastic leukemia (ALL) and associated with increased risk of relapse. Rapid uptake of personal technology makes mobile health (mHealth) an attractive platform to promote adherence.

**Objective:**

Study objectives were to examine access to mobile technology and preferences for an mHealth intervention to improve medication adherence in pediatric ALL.

**Methods:**

A cross-sectional survey was administered in oncology clinic to parents of children with ALL as well as adolescents and young adults (AYAs) with ALL receiving maintenance chemotherapy.

**Results:**

A total of 49 parents (median age [IQR] 39 [33-42] years; female 76% [37/49]) and 15 patients (median age [IQR] 17 [16-19]; male 80% [12/15]) participated. All parents and AYAs owned electronic tablets, smartphones, or both. Parents’ most endorsed mHealth app features included a list of medications (71%, 35/49), information about 6-MP (71%, 35/49), refill reminders (71%, 35/49), and reminders to take 6-MP (71%, 35/49). AYAs' most endorsed features included refill reminders (73%, 11/15), reminders to take 6-MP (73%, 11/15), and tracking 6-MP (73%, 11/15).

**Conclusions:**

Parents and AYAs reported ubiquitous access to mobile technology and strong interest in multiple adherence-specific mHealth app features. Parents and AYAs provided valuable insight into preferred features for a multifunctional behavioral intervention (mHealth app) to promote medication adherence in pediatric ALL.

## Introduction

Acute lymphoblastic leukemia (ALL) is the most common pediatric malignancy [1]. While 50 years ago ALL was invariably fatal, with modern chemotherapy plans, the vast majority of children will achieve remission within 2 months of starting therapy [2,3]. However, further treatment is needed to prevent relapse [2,3]. Despite significant improvements in therapy, 15%-20% of patients still relapse, and cure rates after relapse are considerably less favorable between 20% and 50% [2]. Adding 18-32 months of a low-intensity “maintenance” phase with multiple oral chemotherapy medications, including daily oral 6-mercaptopurine (6-MP), has decreased relapse rates [4].

Low adherence to oral 6-MP has been identified as a significant challenge [5-7], because even minimally suboptimal adherence has been associated with an increased risk of relapse [8-10]. A clinical trial from the Children’s Oncology Group using electronic monitoring devices found that 44% of patients had adherence rates under 95%, which was associated with a 2.7-fold increased risk of ALL relapse compared to those with adherence of 95% or higher [9]. Efforts to promote medication adherence are essential to preventing relapse and improving survival.

Medication adherence is a complex behavior [11]. A variety of interventions have been proposed to improve medication adherence, but behavioral interventions are often complicated and multifaceted with no clear underlying theoretical framework [12]. When these interventions reach their intended goal, disentangling the critical components can be difficult, limiting dissemination [13]. The behavior change wheel (BCW) provides a framework to guide effective development and implementation of behavior change interventions. At the center of the BCW health behaviors are conceptualized in terms of capability, opportunity, and motivation (the COM-B framework) [14,15]. The second layer of the BCW allows researchers to link a conceptualized behavior to 9 intervention functions (education, persuasion, incentivization, coercion, training, enablement, modeling, environmental restructuring, and restrictions). The 9 intervention functions have been mapped to specific behavior change techniques (BCTs) within the Behavior Change Technique Taxonomy (BCTT) [15-17]. The BCTT is a classification system for BCTs developed using a series of expert consensus exercises aimed at improving the reliability and specificity of behavior change interventions by allowing for identification of effective components of behavioral interventions [16]. The BCTT can help researchers identify the active components of interventions that should be implemented to promote a specific element of a behavior. Medication adherence behavior can be better understood and optimized using BCTT.

Mobile health (mHealth) is the use of mobile and wireless applications (eg, SMS text messaging, apps, wearable devices, remote sensing, and the use of social media) in the delivery of health-related services [18,19]. mHealth is an attractive platform for implementing BCTTs because access to mobile technology is widespread. Furthermore, leveraging mHealth interventions is key to optimizing health outcomes in patients with chronic medical conditions during the current COVID-19 pandemic, including ALL [20]. The most recent Pew Research Institute survey reported that 96% of adult US residents own a mobile phone and 81% own a smartphone [21]. Similarly, 95% of teens (age 13-17) reported owning or having access to a smartphone and almost half reported “being online constantly” [22]. The Pew Research Institute survey was designed to capture a sample of households whose demographics mirror the United States nationally but does not necessarily capture individuals with chronic medical conditions. Specifically, families caring for a child with cancer face multiple challenges including material hardship [23] which may affect their access to technology. Ensuring pediatric oncology parents and adolescents and young adults (AYAs) with cancer have access to technology is a prerequisite when considering mHealth interventions.

Access, connectivity, and engagement are all prerequisites for a successful mHealth intervention. Several mHealth apps to promote adherence are available commercially [24] and a pilot study of a medication reminder app demonstrated the feasibility and perceived usefulness of using mHealth in AYAs with cancer [25]. However, mHealth apps have not been yet adopted by pediatric oncology parents [26]. While technology may provide a novel means of implementing behavioral intervention, previous research reports low mHealth use in parents of children with cancer, with Google and WebMD being cited as the most helpful mHealth apps/websites to care for their child [26]. Additionally, while adolescents in general are avid smartphone users, only 2% of teens report frequently using an mHealth app [27]. Being connected to and comfortable with mobile technology are necessary components but are not sufficient for the success of mHealth apps. Successful mHealth apps also need to engage users [28].

Involving users in the development process early promotes engagement [28,29]. User-centered app design is a method for designing mobile apps which starts with a needs assessment followed by iterative cycles involving the intended end user [30]. mHealth apps developed with end user input from the early conceptualization of the intervention through development, deployment, testing, implementation and dissemination are more likely to be perceived by users as useful and easy to use [30]. Engagement in mHealth interventions requires both access to technology and interest in its use [28]. Therefore, understanding patients’ and parents’ access and interest in technology-based (mHealth) interventions is the first step in developing an effective tool to promote medication adherence during maintenance therapy and optimize health outcomes in pediatric ALL.

In this study, we aimed to conduct an mHealth adherence app needs assessment as the first step in user-centered design process. Our first objective was to evaluate access to and comfort with mobile technology among parents who have a child with ALL and AYAs with ALL. Second, we aimed to evaluate interest and preferences for a technology-based (mHealth) intervention (ie, mobile app) to promote adherence to oral chemotherapy during maintenance therapy. We hypothesized that both AYAs with ALL and parents of children with ALL could have variable access to mobile technology and high interest in a mHealth app to promote adherence. We also hypothesized that AYAs with ALL and parents of children with ALL will have different preferences and priorities for mHealth.

## Methods

### Participants and Survey Administration

**Figure 1:**
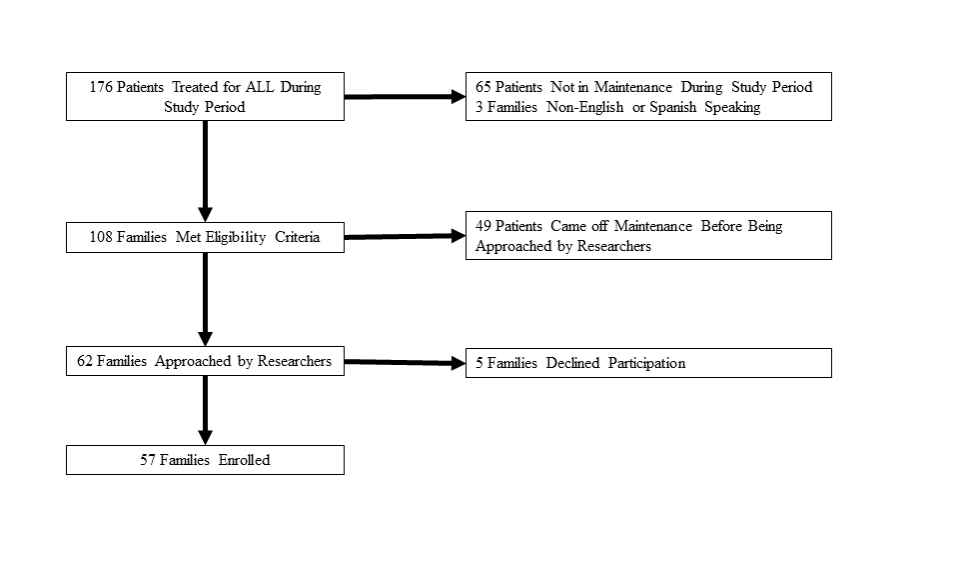
Enrollment flowchart.

We completed a cross-sectional study at a single institution. Eligibility criteria included English or Spanish speaking parents of children with ALL (ages 1-18 years) and patients (ages 12-24 years) with ALL in remission and actively receiving 6-MP as part of the maintenance phase of therapy. Potential study participants were approached before or after regularly scheduled outpatient oncology clinic appointments from November 2017 to March 2019 (Figure 1). Surveys were administered through REDCap using study electronic tablets. Written consent was obtained from all parent participants and patient participants aged over 18. Parent consent and patient assent were obtained for patient participants aged 12-17. The Institutional Review Board at the Ann and Robert H. Lurie Children’s Hospital of Chicago approved the study and all procedures were conducted in accordance with the current version of the Helsinki Declaration.

### Data Collection

Data were collected on electronic tablets through REDCap supported by the Northwestern University Clinical and Translational Sciences (NUCATS) Institute. Medical chart review of enrolled patients was conducted to collect information on ALL disease characteristics and patient characteristics.

### Study Measures

Our study instrument included 65 items assessing technology access, mHealth preferences, and demographics. All portions were developed based on available evidence in the literature related to technology-based interventions and medication adherence for chronic conditions in both adult and pediatric populations that our group has previously published [31-34].

The technology access portion of the survey included 7 questions about access to electronic devices, as well as questions evaluating monthly data plans, SMS text message and call limits, quality and speed of broadband 4G, or home internet signal strength. An additional 10 questions asked about technology use habits and 12 questions assessed participants’ comfort with technology.

The mHealth portion of the survey included 8 yes/no questions, 1 rank order question, and 6 multiple-choice questions that evaluated interest in general mHealth app features and notification preferences, all of which have been reported by our group previously [33]. Our multidisciplinary team of pediatric hematologists, behavioral scientists, and health educators designed these questions, which were later pretested to ensure they were age appropriate for adolescents. Participants were asked to rank the most important features from 1 to 4, with 1 being the most important and 4 being the least important feature. We also developed a list of 20 mHealth features aimed at promoting medication adherence, informed by the Disease Management and Barriers Interview [35], which was developed to identify barriers to adherence, and BCTs [16]. mHealth features were mapped to the BCTT by 2 researchers (MBH and SWC) independently [16,17]. Discrepancies were resolved via discussion with a third researcher (SB), which lead to 100% consensus agreement. According to the BCTT, interventions can have more than 1 function and when agreed upon by 3 researchers (MBH, SWC, and SMB), features were mapped to more than 1 technique. We included the final agreed upon classification of questions in the “Results” section.

### Statistical Analysis

Descriptive statistics for categorical data were reported in frequencies and percentages. Nonparametric stringent statistics were used, because study sample data were not normally distributed. Wilcoxon rank-sum test, Kruskal–Wallis test, chi-square, and Fisher exact tests were performed when appropriate to determine significant association (*P*<.05) among variables. Spearman Rho correlations (r_s_) were calculated to examine the relationship between relevant continuous variables. Statistical analyses were performed using STATA 15.1 (Stata Statistical Software, Release 15.1; StataCorp LP) and MS Excel for Mac 2019 (version 16.27).

## Results

### Participant Characteristics

Of the 62 eligible families approached, 57 (92%) consented to participation (49 parents and 15 patients, including 8 dyads) and completed the study survey (Figure 1). The median age of the participating parents was 39 years (IQR 33-42 years) and 76% were female (37/49). Parents reported on children with a median age of 6 years (IQR 5-10 years). Patient participants had a median age of 17 years (IQR 16-19 years) and 80% (12/15) were male. All participants’ characteristics are summarized in Table 1.

**Table 1:** Participants’ characteristics.

Characteristics		Parent (N=49)	Patient (N=15)
Age, median (IQR)		39 (33-42)	17 (16-19)
**Sex, n (%)**			
	Female	37 (76)	3 (20)
	Male	12 (24)	12 (80)
**Race, n (%)**			
	White	25 (51)	5 (33)
	Black/African American	2 (4)	1 (7)
	Hispanic or Latino	18 (37)	9 (60)
	Asian	4 (8)	2 (13)
**Highest level of education, n (%)**			
	Less than high school	8 (16)	8 (53)
	High-school diploma	6 (12)	5 (33)
	Some college, but no degree	10 (20)	2 (13)
	Associate degree	6 (12)	—
	Bachelor’s degree or higher	19 (39)	—

### Access to and Comfort With Mobile Technology

All parents and patients owned an electronic tablet, a smartphone, or both. Most parents and patients (96% [47/49] and 100% [15/15]) owned smartphones, mainly iPhones (both 73% [36/49, 11/15]), as well as tablets (84% [41/49] and 67% [10/15]) and laptops (63% [31/49] and 67% [10/15]), respectively. Most parents and patients had unlimited plans for SMS text messaging (98% [48/49] and 93% [14/15]) and data (69% [34/49] and 67% [10/15]), as well as a fast home internet connection (92% [45/49] and 93% [14/15]), respectively.

**Figure 2:**
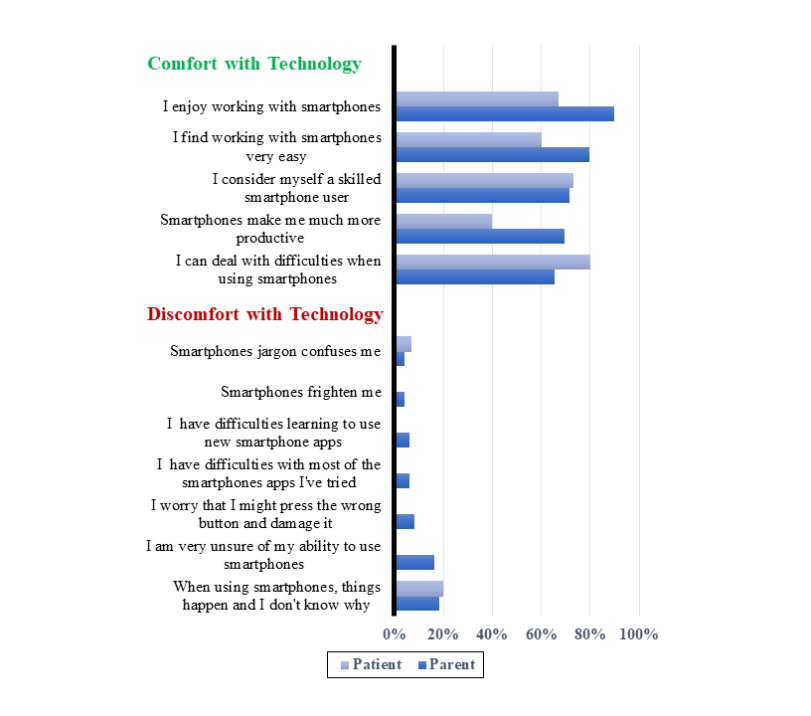
Parents’ and patients’ comfort and discomfort with technology.

Most parents and patients agreed with statements about being comfortable with technology (Figure 2). Only a few parents and patients agreed with statements expressing discomfort with technology. In particular, 18% [9/49] of parents and 20% [3/15] of patients agreed that “When using smartphones things happen, and I don’t know why.” Technology comfort questionnaire data are summarized in Multimedia Appendix 1.

### Interest in mHealth Features

All 8 proposed mHealth features were endorsed by over 50% of parents (25/49) and patients [8/15] (Table 2). The median number of features endorsed by both parents and patients was 7 (IQR 6-8). For parents, the most endorsed features were having the ability to review laboratory results (98%, 48/49), information about ALL medications (96%, 47/49), and information about ALL (92%, 45/49). By contrast, the features most endorsed by patients were daily 6-MP reminders (93%, 14/15), 6-MP tracking (93%, 14/15), and education about ALL (93%, 14/15). Patients were significantly more likely to express interest in 6-MP reminders (93% [14/15] versus 88% [43/49], *P*=.02) and 6-MP tracking (93% [14/15] versus 86% [42/49], *P*=.01), when compared to parents, respectively.

**Table 2:** Frequency of participants’ reported interest in general mHealth features.

Question	Parents (N=49), n (%)	Patients (N=15), n (%)	*P* value
Remind you to take/give your child 6-MP^a^ every day?	43 (88)	14 (93)	.02^c^
Record when you/your child takes 6-MP every day?	42 (86)	14 (93)	.01^c^
Provide encouraging messages when you/your child takes 6-MP?	32 (65)	8 (53)	.87
Send a text message reminder when you/your child hasn’t taken 6-MP?	40 (82)	12 (80)	.69
Virtually connect you to other patients with ALL^b^ and their families?	31 (63)	9 (60)	.16
Provide information about ALL?	45 (92)	11 (73)	.12
Provide information about ALL medications (such as 6-MP and steroid medications) and how they work?	47 (96)	14 (93)	.37
Show the results of your/your child’s blood tests?	48 (98)	13 (87)	.49

^a^6-MP: 6 mercaptopurine.

^b^ALL: acute lymphoblastic leukemia.

^c^*P* value <.05 was statistically significant.

**Figure 3:**
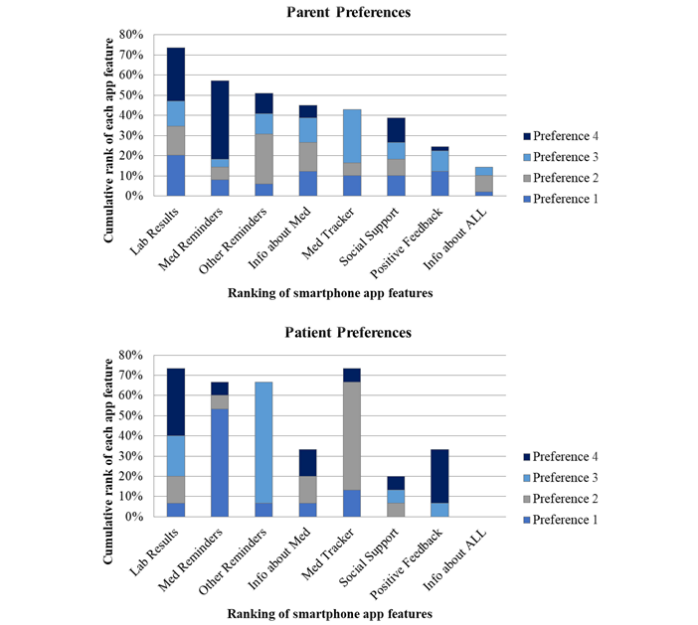
Participants’ cumulative ranking of smartphone adherence app features.

The cumulative ranking of the proposed ALL smartphone app features among parents and AYAs are summarized and illustrated in Figure 3. The ability to review laboratory results (20% [10/49]) was ranked most important most frequently among parents, followed by education about ALL medications (12% [6/49]) and positive feedback (12% [6/49]). By contrast, patients prioritized medication reminders (53% [8/15]) and medication tracker (13% [2/15]).

### mHealth Features to Support Medication Adherence

When asked specifically about their interest in mHealth app features to support medication adherence, parents and patients endorsed a median of 13 features (IQR 8-18) and 11 features (IQR 4-18), respectively (Table 3; *P*=.72). Parents and patients endorsed adherence-promoting app features, mapped to BCTT, which are summarized in Table 3. Parents’ top endorsed adherence-promoting mHealth app features included information about what 6-MP does (71%, 35/49), reminders to refill 6-MP (71%, 35/49), reminders to take 6-MP (71%, 35/49), and a list of medications (71%, 35/49). Patients most frequently expressed interest in reminders to refill 6-MP (73%, 11/15), reminders to take 6-MP (73%, 11/15), and a way to track 6-MP (73%, 11/15). Both parents and patients were most interested in mHealth app features that mapped to prompts and cues (BCTT 7.1). Patients preferred features that utilized self-monitoring behavior (BCTT 2.3), while parents were more interested in instructions on how to perform a behavior (BCTT 4.1) and information about health consequences (BCTT 5.1).

**Table 3:** Participants’ interest in mHealth behavior change techniques focused on promoting medication adherence.

App features by behavior change technique taxonomy (number and label)		Parent (N=49), n (%)	Patient (N=15), n (%)
**1.2.** **Problem solving**			
	Help getting to ALL^a^ appointments	19 (39)	3 (20)
	Help paying for ALL appointments	24 (49)	7 (47)
**2.3. Self-monitoring behavior**			
	A way to track 6-MP^b^ administration	31 (63)	11 (73)
**2.6. Biofeedback**			
	Easier access to my child’s health record	29 (59)	9 (60)
**3.1. Social support**			
	Virtually connect to other patients with ALL and their families	23 (47)	6 (40)
	Information for friends and family about my child’s ALL and 6-MP	26 (53)	8 (53)
**4.1. Instructions on how to perform a behavior**			
	A list of medications on the app	35 (71)	10 (67)
	Information about how to take 6-MP	24 (49)	7 (47)
	Reminders and summaries of doctor’s instructions	34 (69)	9 (60)
**5.1. Information about health consequences**			
	Information about what 6-MP does on an app	35 (71)	10 (67)
	List of side effects of 6-MP on an app	33 (67)	10 (67)
	Information about why it is important to take 6-MP	31 (63)	9 (60)
**7.1. Prompts/cues**			
	App reminders to refill my child’s 6-MP	35 (71)	11 (73)
	App reminders to give 6-MP to my child	35 (71)	11 (73)
	Reminders to take 6-MP when at school or away from home	31 (63)	10 (67)
	Reminders and summaries of doctor’s instructions	34 (69)	9 (60)
**9.1. Credible source**			
	Provide information about ALL?	33 (67)	8 (53)
	Information about what my 6-MP does on an app	35 (71)	10 (67)
	Information for friends and family about my child’s ALL and 6-MP	26 (53)	8 (53)
**12.1. Restructuring the physical environment**			
	An easier way to contact my child’s ALL doctor	25 (51)	7 (47)
	Easier access to my child’s health record	29 (59)	9 (60)
**12.2. Restructuring the social environment**			
	An easier way to contact my child’s ALL doctor	25 (51)	7 (47)
	A way for me to meet other patients with ALL	23 (47)	5 (33)
	A way to contact community centers with ALL resources	25 (51)	7 (47)

^a^ALL: acute lymphoblastic leukemia.

^b^6-MP: 6 mercaptopurine.

## Discussion

Our study contributes to the existing literature on medication adherence in pediatric ALL by reporting on the potential role of mHealth interventions to optimize adherence behavior in this population. Using a cross-sectional survey design, parents and patients with ALL self-reported on their access to technology as well as preferences for mHealth intervention features. We found that all participants owned either a smartphone or an electronic tablet with the majority owning both. Most parents and patients were comfortable using mobile technology. All participants were interested in at least one mHealth feature and all 8 proposed features were endorsed by more than half of both the parent and patient participants. When asked to prioritize these features, parents most commonly rated access to laboratory results, while patients prioritized medication reminders and a medication tracker. Additionally, all parents and patients expressed strong interest in features aimed at promoting medication adherence.

The high rates of technology access are comparable to what has been reported in the general population for AYAs [21,22]. Previous work by Mueller et al [26] demonstrated similar rates of technology access among parents of oncology patients and interest in mHealth [26]. Our study adds to existing literature by demonstrating ubiquitous technology access and interest in mHealth among both parents of children with leukemia and AYAs with leukemia. A unique feature in our study is the increased racial/ethnic diversity in our sample as well as higher completion rate, both of which increase the generalizability of our findings compared to what has been previously reported.

Our findings suggest previously reported low rates of mHealth use by adolescents [27] and parents of children with cancer [26] are not due to a lack of interest in mHealth and represents the first step in user-centered design. Numerous smartphone mHealth apps from commercial vendors are available for use [24,36]. A recent international survey of key stakeholders (ie, clinician, patient organizations, and experts) reported that current mHealth apps seldom meet patients’ expectations and needs because they were not developed with patients in mind [37]. This may account for why most downloaded mobile apps are retained less than a day [38]. Early end user involvement in mobile app development has been associated with increased engagement [28,29]. However, review of the mHealth apps available for download on the iTunes and Google Play app stores confirmed that only a handful of mHealth apps were designed for patients, parents, or both [24]. Further, the majority of these mHealth apps were solely designed for educational purposes, not disease management or medication adherence [24]. Assessing parents’ and patients’ interest in mHealth is the beginning of the continuum of user-centered design. Future efforts should focus on ongoing end user feedback on prototypes and also include features aimed at promoting engagement such as customization, avatars, incentives, and gamification [39].

Previous publications of smartphone mHealth app preferences in pediatric oncology did not focus specifically on medication adherence promotion, which we addressed in this study. Our results are consistent with what has been reported in other complex chronic conditions including cystic fibrosis [40], diabetes [41,42], and sickle cell disease [33], suggesting that a multicomponent smartphone mHealth app may represent a novel intervention to promote oral chemotherapy adherence in patients with ALL. Furthermore, previous systematic reviews demonstrated that interventions with more incorporated BCTs had a larger effect size than other interventions incorporating fewer BCTs [43]. These findings support our patients’ and parents’ interest in multiple key BCTs, which is a unique feature of our study.

AYAs and parents expressed unique priorities. AYAs have different mHealth needs, given their developmental stages and generational differences from their parents. The unique preferences of AYAs are consistent with a review of mHealth interventions suggesting that AYAs may have higher tolerance for SMS text message fatigue than adults, and advocating for adolescent involvement in the development process [32,44]. Parents’ and adolescents’ preferences for an mHealth intervention to promote adherence have not been directly compared previously. However, several studies have found that barriers to adherence vary throughout the lifespan and are unique among adolescents [45,46]. The expressed preference for a medication tracker is consistent with previous studies that suggest organization is important for adolescents. Adolescents expressed low interest in social networking features which is inconsistent with theories on development that suggest adolescents are particularly attuned to peer input and engage in social networks at high rates [46]. In addition, in a qualitative study of adolescents with asthma participants expressed that having the ability to interact with peers on an adherence app would promote adherence. We hypothesize that the lower prevalence of ALL and potential stigma associated with being a patient with pediatric cancer [47] may impact interest in social networking. These differences could be informative and important to consider during a user-centered mHealth app development process to improve engagement with both patients and parents. In other words, having core features for an mHealth ALL app with slightly modified patient and parent versions may be beneficial. Understanding patients’ and parents’ preferences through continued user-centered design approach is vital for the success of mHealth-delivered BCTs [28].

Our study has several strengths. First, we had a high enrollment and completion rate of our survey in a racially/ethnically diverse sample, while previous work evaluating access to technology was limited due to a low response rate. Second, we provide the most comprehensive evaluation of access to technology, evaluating multiple modes of technology as well as comfort with and barriers to technology, including mobile technology, SMS text message, and data plans. Third, we are the first to report on and compare patients’ and parents’ preferences for an ALL mHealth app as a behavioral intervention to promote medication adherence. Finally, we used an established theoretical framework for behavioral intervention development, the BCW/COM-B, and classified our proposed interventions using the BCTT. Using the BCW/COM-B and BCTT allows us to create a targeted intervention while building on previous work in behavior change.

Our study has some limitations worth mentioning. Despite the high enrollment rate and diverse patient population, the generalizability of this study is limited due to data being collected from a single institution with a relatively small sample size. While we adapted validated survey items when possible, not all of our survey items were validated, yet they have been used in other published studies [33,34]. Because we adapted existing survey items, we were not able to provide a more exhaustive list of potential interventions, and we acknowledge that other potential interventions were not included. Finally, we did not ask about current or past use of mHealth apps which could impact participants’ perceptions of mHealth and their preferences.

In conclusion, parents and patients reported ubiquitous access to mobile technology and high levels of interest in general ALL mHealth app features and features intended to promote medication adherence. Our findings highlight the need for the development of a user-centered mHealth intervention (mobile app) to promote medication adherence among children and AYAs with ALL and their parents. Designing 2 separate app versions for each group of end users, parents and patients, may be needed to optimize engagement with the app as an intervention and improve care delivery, and ultimately health outcomes in this vulnerable population of pediatric ALL.

### Data Availability Statement

The data sets generated during or analyzed during this study are available from the corresponding author on reasonable request.

## Appendix

Multimedia Appendix 1 Participants’ comfort and discomfort with mobile technology.

## Abbreviations

6-MP: 6-mercaptopurine
ALL: acute lymphoblastic leukemia
AYAs: adolescents and young adults
BCT: behavior change technique
BCTT: Behavior Change Technique Taxonomy
mHealth: mobile health
NUCATS: Northwestern University Clinical and Translational Sciences
