# Supplementary material for: Access to Technology and Preferences for an mHealth Intervention to Promote Medication Adherence in Pediatric Acute Lymphoblastic Leukemia: Approach Leveraging Behavior Change Techniques
Source: J Med Internet Res. 2021 Feb 18;23(2):e24893. doi: 10.2196/24893 (PMC7932843; doi:10.2196/24893)
Supplement: Multimedia Appendix 1 [file jmir_v23i2e24893_app1.docx]

**Multimedia Appendix 1. Participants’ comfort and discomfort with mobile technology**

|  | **Parent** | **Patient** |
| --- | --- | --- |
| **Comfort with technology, n (%)** |  |  |
| I can deal with difficulties when using smartphones | 32 (65%) | 12 (80%) |
| Smartphones make me much more productive | 34 (69%) | 6 (40%) |
| I consider myself a skilled smartphone user | 35 (71%) | 11 (73%) |
| I find working with smartphones very easy | 39 (79%) | 9 (60%) |
| I enjoy working with smartphones | 44 (90%) | 10 (67%) |
| **Discomfort with technology, n (%)** |  |  |
| I am very unsure of my ability to use smartphones | 8 (16%) | 0 (0%) |
| I worry that I might press the wrong button and damage it | 4 (8%) | 0 (0%) |
| I have difficulties with most of the smartphones apps I've tried | 3 (6%) | 0 (0%) |
| I have difficulties learning to use new smartphone apps | 3 (6%) | 0 (0%) |
| Smartphones frighten me | 2 (4%) | 0 (0%) |
| Smartphones jargon confuses me | 2 (4%) | 1 (7%) |
| When using smartphones, things happen, and I don't know why | 9 (18%) | 3 (20%) |
